# Time-Dependent Association Between Cardiotocographic Features and Hypoxic-Ischemic Encephalopathy

**DOI:** 10.1109/access.2025.3570371

**Published:** 2025-05-15

**Authors:** JOHANN VARGAS-CALIXTO, YVONNE W. WU, MICHAEL KUZNIEWICZ, MARIE-CORALIE CORNET, HEATHER FORQUER, LAWRENCE GERSTLEY, AARON W. SCHEFFLER, PHILIP A. WARRICK, ROBERT E. KEARNEY

**Affiliations:** 1 Department of Biomedical Engineering, McGill University, Montreal, QC H3A 2B4, Canada; 2 Department of Neurology, University of California at San Francisco, San Francisco, CA 94158, USA; 3 Department of Pediatrics, University of California at San Francisco, San Francisco, CA 94143, USA; 4 Division of Research, Kaiser Permanente Northern California, Oakland, CA 94612, USA; 5 Department of Epidemiology and Biostatistics, University of California at San Francisco, San Francisco, CA 94158, USA; 6 PeriGen Inc. Medical Research, Cary, NC 27518, USA

**Keywords:** Acidosis, classification, fetal heart rate, hypoxic-ischemic encephalopathy, labor and delivery, machine learning, mutual information

## Abstract

Neonatal hypoxic-ischemic encephalopathy (HIE) is caused by sustained hypoxemia near birth. Clinical assessment using cardiotocography (CTG), which measures the fetal heart rate (FHR) and maternal uterine pressure (UP), aims to identify infants at increased risk of HIE. Although CTG is nonstationary, current automated methods for its analysis use time invariant discrimination rules. Our objective was to examine the association between features of CTG and the development of HIE to determine if accounting for the time to delivery (TTD) would strengthen these associations. We analyzed 88 features extracted from FHR and UP signals from 25,197 vaginally delivered infants for whom blood gas measurements were available. All infants were categorized according to their blood gas exams into three mutually exclusive groups: 167 HIE, 1,912 acidosis - a precursor to HIE, and 22,903 healthy cases. We evaluated CTG features during the last twelve hours of labor to explore the associations between 1) CTG features and TTD, 2) CTG features and the development of HIE, and 3) the conditional association between CTG features and the development of HIE given TTD. These associations were quantified using the normalized mutual information. We found that all CTG features varied with TTD. Furthermore, 48 out of 88 features were not significantly associated with the outcome of labor and might not be useful in classification studies. We also found that 40 out of 88 features had significant associations with the development of HIE; accounting for TTD increased the association for 26 of these features. Therefore, automated methods for prediction of infants at risk of HIE should focus on this set of CTG features and account for their time-varying properties.

## I. INTRODUCTION

Neonatal hypoxic ischemic encephalopathy (HIE) is a syndrome of disturbed neurologic function appearing in the earliest days of life resulting from sustained hypoxemia around the time of birth. Although its incidence is relatively low in developed countries (1.3 to 1.7 cases per 1,000 live births), it carries a very heavy burden in developing countries where the incidence is much higher (6.7 to 26.5 per 1,000 live births) [1], [2]. Moderate and severe forms of HIE are associated with an increased risk of neonatal death (12% to 31%) [2] and permanent impairments such as cerebral palsy, motor disorders, cognitive disorders, and learning disabilities (25% to 30%) [3].

Direct measurement of cerebral oxygen levels or fetal blood pressure to detect hypoxemia is not feasible during labor. Instead, clinicians use cardiotocography (CTG), the ensemble of fetal heart rate (FHR) and uterine pressure (UP) signals, to indirectly assess when fetuses are at an increased risk of developing HIE. As labor progresses, uterine contractions become more frequent and intense. These limit the exchange of gases in the placenta resulting in intermittent periods of fetal hypoxemia. Fetal chemoreceptors respond to low oxygen and pH in fetal circulation, and baroreceptors respond to changes in arterial blood pressure. These receptors bring about changes in the FHR and in the distribution of blood flow to optimize oxygen delivery to critical organs and restore fetal homeostasis. Therefore, clinicians use CTG to try to detect the changes in FHR associated with severe fetal hypoxia.

Obstetrical societies have defined graded classifications based on specific combinations of CTG patterns to help clinicians assess the fetal risk of hypoxic injury and guide clinical management [4]. This classification depends on the correct identification of the baseline, accelerations, and decelerations in the FHR, as well as contractions in the UP signal. As an example, Table 1 shows the guidelines provided by the National Institute of Child Health and Human Development (NICHD) for the interpretation of intrapartum FHR [4]. Other societies, such as the International Federation of Gynecologists and Obstetricians (FIGO) provide similar classifications [5]. Although not explicitly included in Table 1, uterine contractions are considered to increase the risk of fetal hypoxia when they occur more than 15 times in a 30-minute period, a condition known as tachysystole [4], [5].

Although obstetrical guidelines are based on the interpretation of physiological FHR responses to hypoxia-inducing stimuli, these classifications have significant problems: the visual identification of basic FHR patterns is inconsistent [6], [7], classification systems do not specify in detail how the evolution of patterns should be considered, and none has a sufficiently high sensitivity to detect HIE with acceptable specificity [8], [9], [10], [11].

Efforts have been made to improve the consistency of the FHR analysis using computerized signal processing in the hope that better feature measurements would improve the graded classification of tracings [12]. This has led to the proliferation of potential features with little understanding of how informative they might be. Hence, it is important to determine which predictors are likely to be most informative in HIE studies.

While changes in CTG patterns throughout labor are expected, there is a need for observational studies to assess how CTG features evolve [4]. To date, most CTG classification studies have used time-invariant (TI) models, or models that were trained using data only from the last 60 to 90 minutes of labor without accounting for CTG evolution [13], [14], [15], [16]. Furthermore, most CTG classification models have been trained to consider moderate fetal acidosis as a surrogate for severe fetal pathology, due to the low availability of HIE cases [15], [16], [17]. However, it is important to quantify the associations among CTG features and clinically relevant labor outcomes such as HIE [4].

The goal of the present study was to evaluate the hypothesis that CTG features vary with time, i.e. they are nonstationary. Furthermore, it aimed to evaluate the association between these features and the development of HIE in labor, and the importance of accounting for CTG evolution using time-varying (TV) methods. To do so, we quantified associations of 88 selected CTG features using mutual information. Unlike Pearson’s correlation, mutual information identifies both linear and nonlinear dependencies [18] and has been used in machine-learning studies to quantify predictor importance [19]. Our analysis used a large database of CTG recordings from as early as 12 hours before delivery. We quantified the associations with outcome of labor defined by three mutually exclusive groups: (1) infants that developed HIE, (2) infants that developed acidosis - a precursor to HIE, and (3) healthy infants.

## II. METHODS

### A. STUDY POPULATION

Clinical and CTG data, from as early as 72 hours before delivery, were collected from 297,280 births across 15 Kaiser Permanente Northern California (KPNC) hospitals between 2011 and 2019. Only singleton live births with a gestational age of at least 35 weeks, having no major congenital or chromosomal anomalies were included.

The Research Ethics Boards (REB) of Kaiser Permanente (approval numbers 1470283–5 and 1276201–29) and McGill University (approval numbers A04-M27–20A (20–04-025) and A04-M29–20B (20–04-056)) reviewed the study. Both REBs concluded that the study involved secondary research of data for which consent was not required.

**FIGURE 1.**
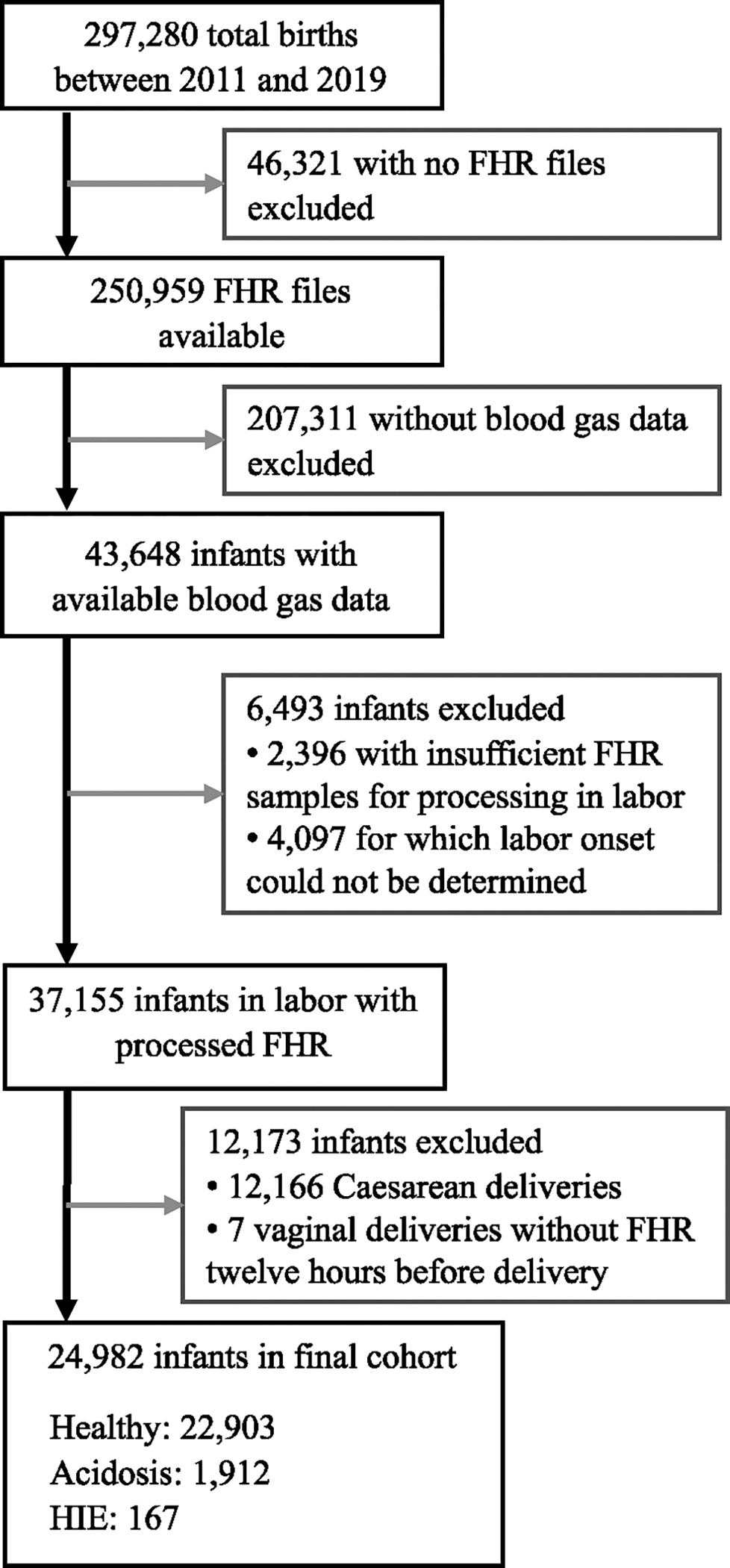
Inclusion and exclusion criteria of the infants included in the study.

Fig. 1 shows the inclusion and exclusion criteria for the study population. The analysis was limited to mother-infant dyads that had blood-gas data available. The blood-gas results were used to determine acidosis at birth, which is a marker of HIE. We analyzed data as early as 72 hours before delivery to determine the onset of labor [20]. We discarded all cases where labor onset could not be identified, and we removed all signal content occurring before labor onset. Then, we limited the analysis to the last 12 hours of labor. All fetuses that did not have data available in this period were removed from analysis.

We excluded from analysis those fetuses that underwent Caesarean delivery. The goal of this study was to understand how the CTG features are related to the time to delivery (TTD) and to the outcome of labor. Including Caesarean deliveries could bias the results since the time of delivery is determined by the clinician and not by the natural end of labor. Also, while in this study we used the TTD to quantify the time-varying behavior of the CTG features, this variable is not available prospectively for real-time classification. The goal of using TTD in this study is to show that the features are time-varying, and that accounting for this could be beneficial to gain information about the outcome of labor. We believe that the TTD is useful for this objective, and finding the best time variable that is available prospectively could be the focus of future studies.

The study population was divided into three mutually exclusive outcome groups based on the blood-gas results and clinical data [20], [21]:

1. The intrapartum HIE group (n = 167) had acidosis at birth and neonatal encephalopathy (NE). Acidosis at birth was defined by a cord-gas pH < 7.0 or base deficit (BD) ≥ 10 mmol/L, or neonatal-gas BD ≥ 10 mmol/L. While blood pH measures blood acidity, BD measures the depletion of the bicarbonate reserves in fetal blood, and it is associated with metabolic acidosis. NE was determined by a documented abnormal neurological examination that was performed within the first six hours of life. In addition, one of the following clinical indications had to occur: the abnormal neurological exam persisted beyond six hours of life, neonatal seizures occurred during the first 24 hours of life, or the infant was treated with active therapeutic hypothermia. All infants suspected of having developed HIE underwent a chart review by a panel of clinicians as previously described [21].
2. The acidosis group (n = 1,912) presented acidosis at birth but no evidence of encephalopathy. Acidosis at birth was defined by the same criteria as for the HIE group. This group did not receive therapeutic hypothermia, had no abnormal neurological exams within six hours of birth, and had no seizures in the first 24 hours of life.
3. The healthy group (n = 22,903) had cord-gas pH ≥ 7.0 and BD < 10 mmol/L, or neonatal-gas BD < 10 mmol/L. The Apgar score at five minutes after birth had to be ≥ 7. This group were never admitted to the neonatal intensive care unit, were discharged home, had no deaths or hospital transfers, had no evidence of encephalopathy, did not require intubation or chest compression during newborn resuscitation, had no evidence of seizures, did not receive seizure medications, and did not require therapeutic hypothermia.

### B. FHR PREPROCESSING

The FHR signal was sampled at 4 Hz. We used PeriCALM Patterns, a software system developed by PeriGen Inc., to preprocess the FHR. PeriCALM Patterns identified gaps in the signal and segments with high noise levels were labeled as uninterpretable. It filled gaps shorter than 60 samples (15 s) using linear interpolation. PeriCALM Patterns filtered the FHR using low-pass, high-pass, median filters, and a Karhunen-Loève filter [22], [23]. Recordings that could not be preprocessed due to having very few samples or being mostly uninterpretable were removed from analysis.

After preprocessing, PeriCALM Patterns used a long-short-term memory network to identify the location and duration of the CTG events that are the focus of clinical FHR assessment [22], [23]. These included:

- FHR baselines: relatively flat segments usually between 110 and 160 bpm with a peak-to-peak variability between 5 and 15 bpm.
- FHR accelerations: segments where the FHR increased by more than 15 bpm from the baseline for more than 15 seconds before returning to baseline.
- FHR decelerations: segments where the FHR decreased by more than 15 bpm from the baseline for more than 15 seconds before returning to baseline.
- Uterine contractions: were identified from the UP signal. These were indicated by sudden increases in uterine pressure followed by a return to resting level.

**FIGURE 2.**
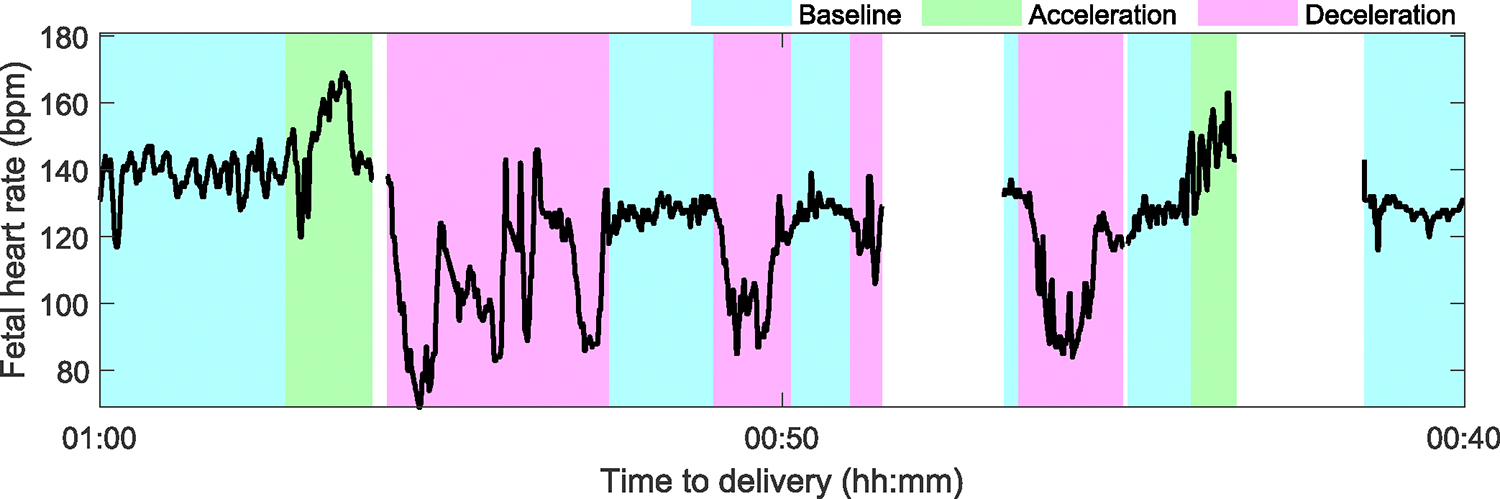
FHR patterns of an epoch that started 1 hour before delivery and ended 40 minutes before delivery. The FHR belongs to a healthy infant. PeriCALM Patterns identified the FHR baseline (cyan), accelerations (green), and decelerations (magenta). The blank spaces correspond to gaps and uninterpretable FHR segments which were removed from analysis.

The FHR signals were divided into non-overlapping 20-minute epochs for further analysis. Epochs with more than 20% of samples missing or labeled as uninterpretable were not considered for further analysis. Fig. 2 shows one epoch of an FHR tracing from an infant with a healthy outcome. Our methods paper provides a more detailed description of CTG preprocessing and event identification [20].

### C. EXTRACTION OF CTG FEATURES

Table 1 described the FHR properties used in clinical practice. We extracted seven clinically relevant features from the FHR and UP epochs for up to 12 hours before delivery:

- Baseline level: The mean value of the baseline segments within each epoch.
- Baseline variability: Obtained from the standard deviation of the FHR during baseline segments after high-pass filtering. The FHR was high-pass filtered to remove the mean and trend before computing its variability [20]. Assuming a normal distribution, the peak-to-peak variability of the baseline could be interpreted as 3.92 times its standard deviation.
- Number of late decelerations per epoch.
- Number of variable decelerations per epoch.
- Number of decelerations per epoch that were neither late nor variable.
- Number of accelerations per epoch.
- Number of uterine contractions per epoch.

Many novel CTG time-domain, frequency-domain, and nonlinear features have been proposed for the detection of fetal acidosis during the intrapartum, as discussed in several review papers [24], [25]. We extracted the majority of these from each FHR epoch. The complete list of features extracted is provided in the Appendix A, and in our methods paper [20].

For each FHR epoch, we extracted 26 features from the baseline segments, 28 from acceleration segments, 31 from deceleration segments; from the UP signal we extracted 3 features. This gave a total of *N*_feats_ = 88 CTG features per epoch, including the clinical features described above. Segmenting the FHR into baseline, acceleration, and deceleration regions, prior to feature extraction in such a large database, is a novelty of our work. This was possible due to automatic segment labeling using PeriCALM Patterns.

### D. MUTUAL INFORMATION

We used mutual information to measure the linear and nonlinear associations between variables [18]. Mutual information is closely related to entropy in information theory. The entropy H(X) of a discrete random variable X quantifies its uncertainty and is related to its variability [18]. It is defined from the probability mass function p(x) as

(1) $$H\left(X\right)=-\sum_{x\in X}p\left(x\right)\log\left(p\left(x\right)\right).$$

The mutual information I(X;Y) between two discrete variables X and Y with joint probability mass function p(x,y), and marginal probability mass functions p(x) and p(y) is defined as

(2) $$I(X;Y)=\sum_{x\in X}\sum_{y\in Y}p(x,y)log\left(\frac{p(x,\;y)}{p(x)p(y)}\right)$$

estimated across all possible values of X and Y [18], [26]. This equation implies that I(X;Y)=H(X)-H(X∣Y), so mutual information quantifies how much the uncertainty about X is reduced by knowledge of Y. If X and Y are independent, then I(X;Y)=0.

The mutual information can be normalized by the entropy of either variable for feature comparisons. Given that I(X;Y)≤H(X), the normalized mutual information (NMI) is bounded by

(3) $$0\leq {NMI}_{X}(X;Y)=\frac{I\left(X;\;Y\right)}{H\left(X\right)}\leq 1.$$

For three variables, the conditional mutual information quantifies the advantage of accounting for a third variable inthe study of the association of two variables. Thus, I(X;Y∣Z) describes the mutual information between X and Y when accounting for Z; replacing Y with the conditional in (2) gives

(4) $$I\left(X;Y|Z\right)=\sum_{x\in X}\sum_{y\in Y}\sum_{z\in Z}p\left(x,y,z\right)\log\left(\frac{p\left(z\right)p\left(x,\;y,\;z\right)}{p\left(x,\;z\right)p\left(y,\;z\right)}\right).$$

This makes it evident that if X and Y are independent of Z, then I(X;Y∣Z)=I(X;Y). In contrast, if Y is perfectly correlated with Z, then I(X;Y∣Z)=0 because Y provides no new information once Z is given. When I(X;Y∣Z)>I(X;Y), accounting for Z increases the association between X and Y [27]. The conditional NMI can be defined as

(5) $$0\leq {NMI}_{X}(X;Y|Z)=\frac{I\left(X;\;Y|Z\right)}{H\left(X\right)}\leq 1.$$

to assess the reduction in the uncertainty of X by the knowledge of Y when in a Z-varying analysis.

The entropy and NMI of continuous variables can be approximated from their discretized empirical distribution using binned histograms [18], [26]. Some of the CTG features we computed were discrete (e.g. number of contractions per epoch) but many had continuous distributions. It was important to choose an appropriate number of bins to discretize these continuous variables. Too few bins would produce a biased estimate of the distribution; too many bins would increase the random error of the estimates. We used Rice University’s discretization rule [28], which is based on the number of available samples, to select the number of bins for two reasons: (1) we had a limited number of samples in the HIE group compared to the other two groups, and (2) we needed a consistent number of bins across all continuous variables to allow for comparability of their NMI.

Rice University’s discretization rule defines

(6) $$N_{bins}\approx 2\sqrt[{3}]{N_{samp},}$$

where $N_{\text{bins}}$ is the minimum number of bins needed to discretize the whole range of a variable, and $N_{\text{samp}}$ is the number of samples available for that variable [28]. In total, there were 4,040 epochs coming from HIE cases. This number of samples resulted in $N_{\text{bins}}\approx 31.85$. Thus, we selected $N_{\text{bins}}=32$ to discretize the range of the continuous variables. Next, we used $N_{\text{bins}}$ to define feature-dependent quantization levels w as:

(7) $$w\left(Y\right)=\frac{P_{99}\left(Y\right)\;-\;P_{1}\left(Y\right)}{N_{bins}},$$

where $P_{n}(Y)$ is the nth percentile of Y. We defined the range of the variable to be the distance between the first and 99th percentiles, to avoid defining bins that are too wide for variables whose distribution had long tails. The resulting complete set of discrete features F, the time to delivery TTD, and infant category C (healthy, acidosis, or HIE) were used to obtain the empirical distributions used for mutual information estimation.

### E. ASSOCIATIONS AMONG CTG FEATURES, TIME, AND INFANT GROUPS

We evaluated the following associations among variables using their mutual information:

- The association of each feature with time ${NMI}_{F^{i}}\left(F^{i};TTD\right)$ we quantified the dependency of the *i*th CTG feature $F^{i}$, where $1<i\leq N_{\text{feats}}$, on the time to delivery, TTD. This statistic was used to test if the features were time varying.
- The association of the features on the outcomes of labor ${NMI}_{C}\left(C;F^{i}\right)$: we quantified the static association between a CTG feature and the infant groups, described by the categorical variable C. The computation was performed without accounting for the TTD. This statistic was used to test whether CTG features could be useful in time-invariant (TI) classification studies.
- The conditional association between features and outcomes of labor when TTD is known $NMIC\left(C;F^{i}|TTD)\right.$: we quantified the association between the CTG features and the infant groups when accounting for TTD. This statistic was used to test if the classification of infant groups using time-varying (TV) CTG features was superior to TI-based models.

### F. PERMUTATION TESTS

We used permutations tests to assess the significance of the null hypotheses (NH) [29]. This test approximates the distribution of the NH making no assumptions. We estimated the significance of our statistics by comparing their values to the NH distributions. Exact permutation-based tests require evaluating all $N_{\text{perm}}$ possible combinations of the variables, but they can be approximated by Monte Carlo sampling [30]. This consists of drawing $N_{\text{MC}}\ll N_{\text{perm}}$ independent samples to estimate the distribution generated by full permutation. Thus, we needed to select a number of permutations that was smaller than the number of all possible permutations, but large enough to make it possible to reject the null hypotheses. Previous studies have demonstrated that $N_{MC}=5,\;000$ realizations give stable p-values for a desired rejection level of p<0.05 [31]. Then, we performed two types of permutations:

- Permuting the C variable across all infants to break the association between CTG features and the infant groups. This generated $H_{0}\left({NMI}_{C}\left(C;F^{i}\right)\right)$, the distribution of the NH that the features are not related to the outcome of labor. This permutation was stratified by the hospital to maintain intra-hospital variability.
- Permuting the TTD variable, stratified within each infant, to break the association between CTG features and time. This generated $H_{0}\left({NMI}_{F^{i}}\left(F^{i};\right.TTD\;\left.)\right)\right.$, the distribution of the NH that the CTG features do not depend on time. This also generated $H_{0}\left({NMI}_{C}\left(C;F^{i}|TTD\right)\right)$, the distribution of the NH that accounting for time does not improve the association of CTG features with the infant group.

We compared S, the set of NMI estimates of all $N_{feats}$ features, to their NH distributions $H_{0}(S)$. The significance level of the tests were defined as the proportion of NH samples that were greater or equal to S:

(8) $$p=\frac{1}{N_{MC}}\sum_{i=1}^{N_{MC}}\left(H_{0}(S)\geq S\right)$$

where p is the array of significance levels for all tests in S.

In total, we performed three NH tests using permutations for each CTG feature. Thus, the array p contained the results of $3N_{feats}=264$ tests. Multiple comparisons using the same dataset increase the risk of type I error. To account for this, we used the Benjamini and Hochberg (BH) method to correct the values in p [32], [33]. This method controls the false discovery rate (FDR), the proportion of the rejected null hypotheses that were in fact true. After correction, we rejected all null hypotheses that had a significance level smaller than 0.05.

Based on their associations between the infant groups, we categorized the CTG features as follows:

1. Type I features had a significant TI association with the infant groups but no TV association. Differences in these features among groups do not vary with time, so their discriminability does not vary with TTD.
2. Type II features had a TV association with the infant group but no TI association. These features would be discarded by TI classifiers. However, accounting for time would make them discriminative.
3. Type III features had significant TI and TV association with the infant groups. Accounting for TTD would improve discriminability.
4. Null features had no significant TI or TV association with the infant groups. These features would not be useful in classification studies.

### G. BINARY DISCRIMINABILITY

To complement the NMI results, we assessed the ability of each TI and TV feature to discriminate the healthy class from a pathological class, which included the combination of the acidosis or HIE groups, using the two-sample Kolmogorov-Smirnov statistic.

Given the difference $d_{P}(x)=\left|P_{1}(x)-P_{2}(x)\right|$ between two cumulative distributions P(x) of two samples of the same variable x, the KS statistic is the maximum of the difference $d_{KS}(x)={max}_{x}\left(d_{P}(x)\right)$. The $d_{KS}$ function quantifies the discriminability of a variable and is directly related to the area under the receiver-operating characteristics curve [34]. Therefore, $d_{KS}$ is a model-free method to evaluate feature importance. Furthermore, the value of the variable x for which the distance between two distributions is maximal is the classification threshold that provides the greatest separability of the two classes.

We compared TI and TV estimates of the KS statistics, $d_{KS}\left(F^{i}\right)$ and $d_{KS}\left(F^{i}|\right.TTD=t)$, for each CTG feature and for all values of t∈TTD. We also computed $\overset{ˆ}{f_{TI}}=arg\;max\left(d_{KS}\left(F^{i}\right)\right)$, the $F^{i}$ value with the greatest distance between healthy and pathological distributions. Similarly, for all t∈TTD, we computed $F^{i}$ values $\overset{ˆ}{f_{TV}}(t)=arg\;max\left(d_{KS}\left(F^{i}|TTD=t\right)\right)$.

We used bootstrapping to estimate the variability of the estimated KS statistics and thresholds. Thus, we randomly sampled, with replacement, from the healthy and pathological classes for 10,000 iterations and computed the corresponding statistics for each iteration.

## III. RESULTS

### A. CHARACTERISTICS OF THE STUDY POPULATION

Table 2 shows the medians, and interquartile ranges of some clinical and demographic characteristics of the participants. We compared each pathological group to the healthy group. The acidosis and HIE groups were not different from the healthy group in terms of birth weight. Gestational age was significantly different in healthy versus acidosis groups. However, Cohen’s *d* effect size showed that the difference was small. The HIE group showed no differences from the healthy group in terms of gestational age. Maternal age was different from the healthy group only for the acidosis group, but the effect size was small. Maternal weight was different for both pathological groups when compared to the healthy group but, the effect sizes were small in both cases. The number of nulliparous pregnancies was quite different across the three groups. Compared to healthy cases, nulliparity was more frequent for the HIE and acidosis groups; both differences were statistically significant. Finally, labors were significantly longer in the pathological groups than in the healthy groups. The effect size was greater for the HIE-healthy comparison than for the acidosis-healthy comparison.

### B. AVAILABILITY OF FHR TRACINGS

**FIGURE 3.**
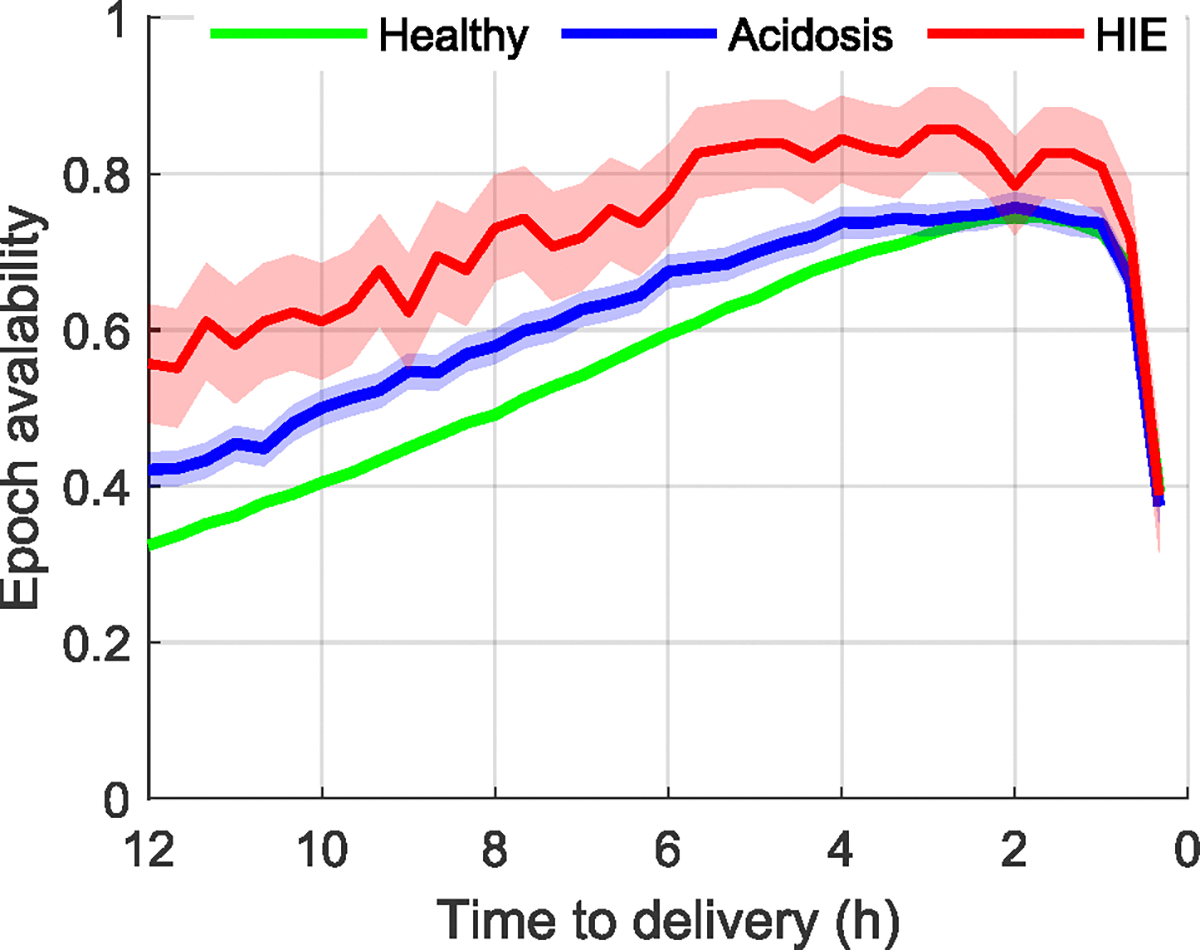
Epoch availability for the three infant groups during the last 12 hours of labor. The solid lines indicate the proportion of infants from each group that had CTG data available at each time, the shaded area indicates the 95% confidence interval of this estimate.

All infants included in this study had at least one epoch available during the last 12 hours of labor. Fig. 3 shows the proportion of infants from each group with epochs available as a function of time during the last 12 hours of labor. For all three groups, epoch availability increased progressively as delivery approached and then dropped dramatically in the last hour. At all times, the HIE group had a greated proportion of FHR epochs available than the acidosis or the healthy groups.

### C. OVERVIEW OF ASSOCIATIONS

**FIGURE 4.**
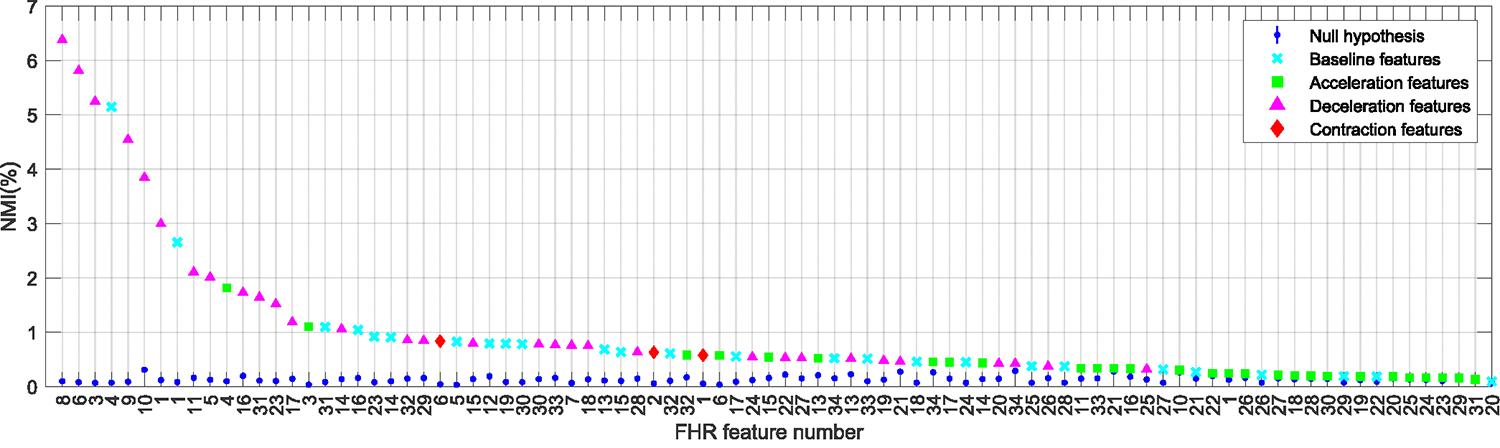
Time-varying features. Normalized mutual information (NMI) between FHR features that rejected the null hypothesis, and the time to delivery (TTD). The features were extracted from the FHR baseline (cyan), acceleration (green), deceleration (magenta), and contraction events. The range of acceptance of the null hypotheses is indicated in blue. See Table 5 for the names of each feature number.

**FIGURE 5.**
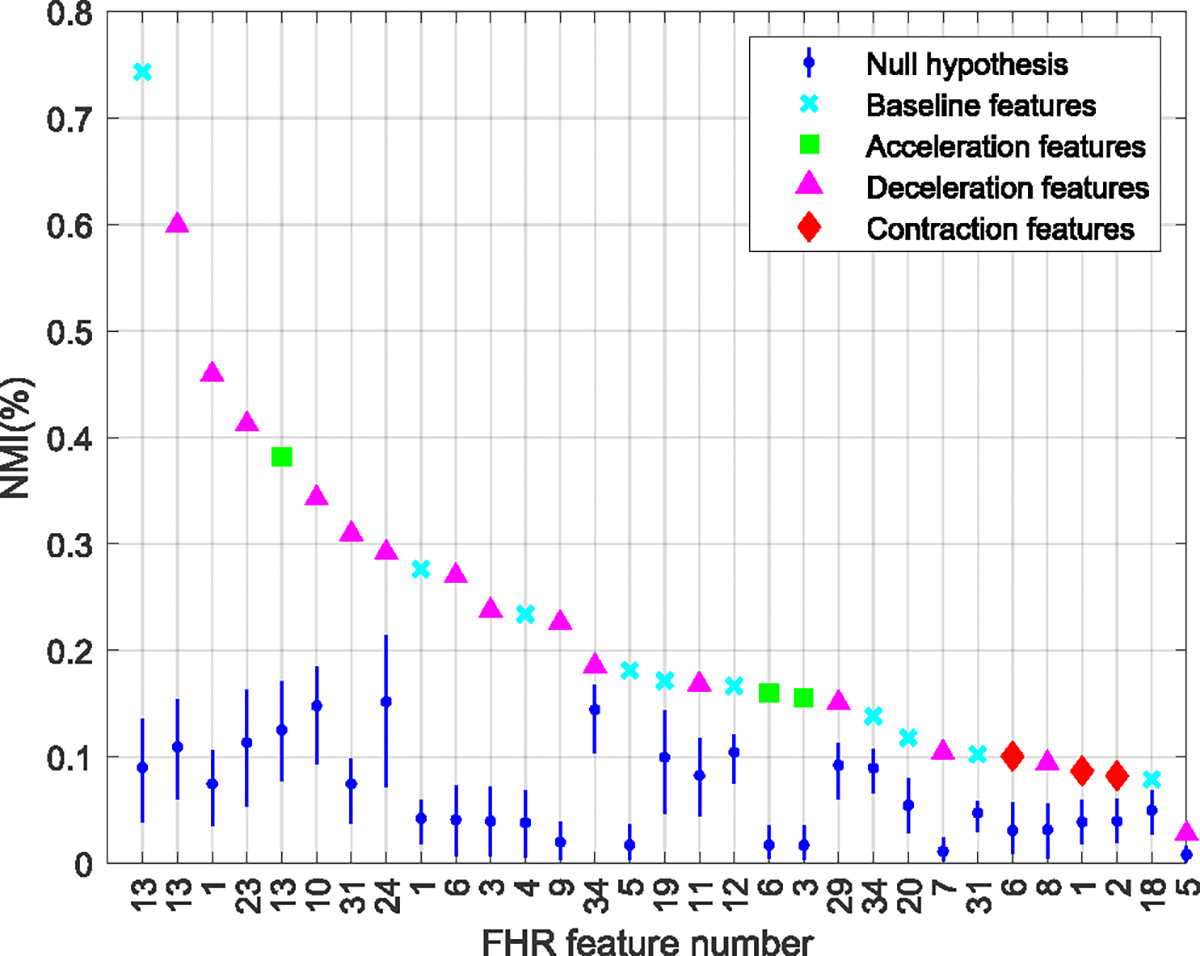
Features with a time-invariant association with outcome class. Normalized mutual information (NMI) between FHR features that rejected the null hypothesis, and the outcome of labor. The features were extracted from the FHR baseline (cyan), acceleration (green), deceleration (magenta), and contraction events. The range of acceptance of the null hypotheses is indicated in blue. See Table 5 for the names of each feature number.

**FIGURE 6.**
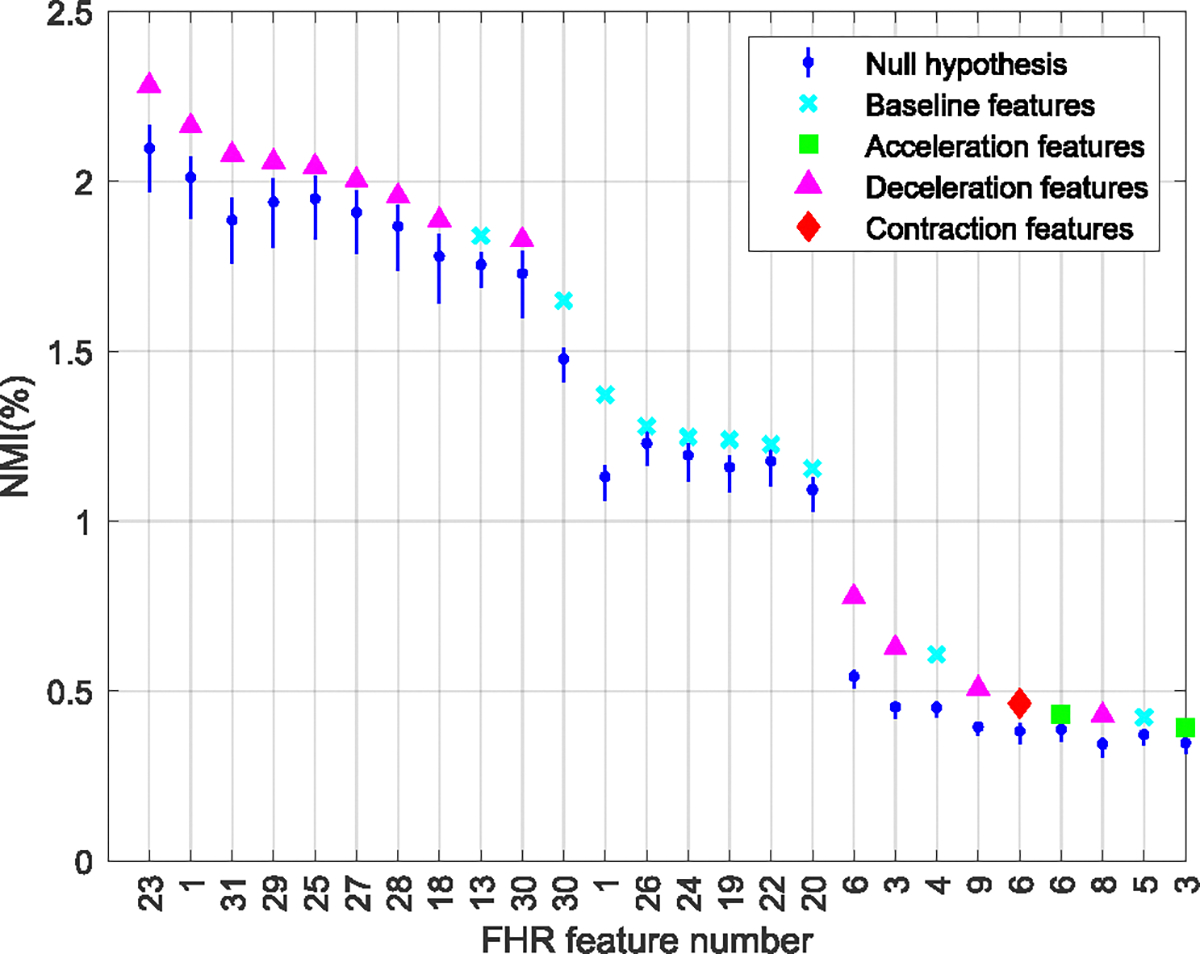
Features with a time-varying association with outcome class. Normalized mutual information (NMI) between FHR features that rejected the null hypothesis, and the outcome of labor conditioned on time to delivery (TTD). The features were extracted from the FHR baseline (cyan), acceleration (green), deceleration (magenta), and contraction events. The range of acceptance of the null hypotheses is indicated in blue. See Table 5 for the names of each feature number.

Figs. 4, 5, and 6 summarize the NMI estimates for TV features, TI features associated with outcome class, and TV features associated with outcome class. Only features that rejected the null hypothesis at the 95% confidence are shown. Table 3 lists the null features, those who did not have any significant association with the outcome of labor.

Fig. 4 shows ${NMI}_{F^{i}}\left(F^{i};TTD\right)$, the NMI between the FHR features and TTD, for features sorted in decreasing order of their association with TTD. All features showed a significant association with TTD and were thus TV. Table 5 gives the names of each feature number on the horizontal axis (Appendix A). The features with the highest association with TTD came from deceleration and baseline events, while most of the features with low association came from acceleration events.

Fig. 5 shows ${NMI}_{C}\left(C;F^{i}\right)$, the NMI between the TI features and the infant groups. Only 31 out of 88 features had a significant TI association with the infant groups. These comprised 10 baseline, 3 acceleration, 15 deceleration, and all three contraction-based features.

Fig. 6 shows ${NMI}_{C}\left(C;F^{i}|TTD\right)$, the NMI between the features and the infant group conditioned on TTD. Here, only 26 out of the 88 features showed a significant increase in their association with the infant groups by accounting for TTD. These included 10 baseline, two acceleration, 13 deceleration, and one contraction-based features.

### D. ASSOCIATION AMONG CTG FEATURES, TIME, AND INFANT GROUPS

Table 3 lists the 40 null features, those that showed no significant TI or TV association with the outcome groups, indicating that they would have little utility for classification.

**FIGURE 7.**
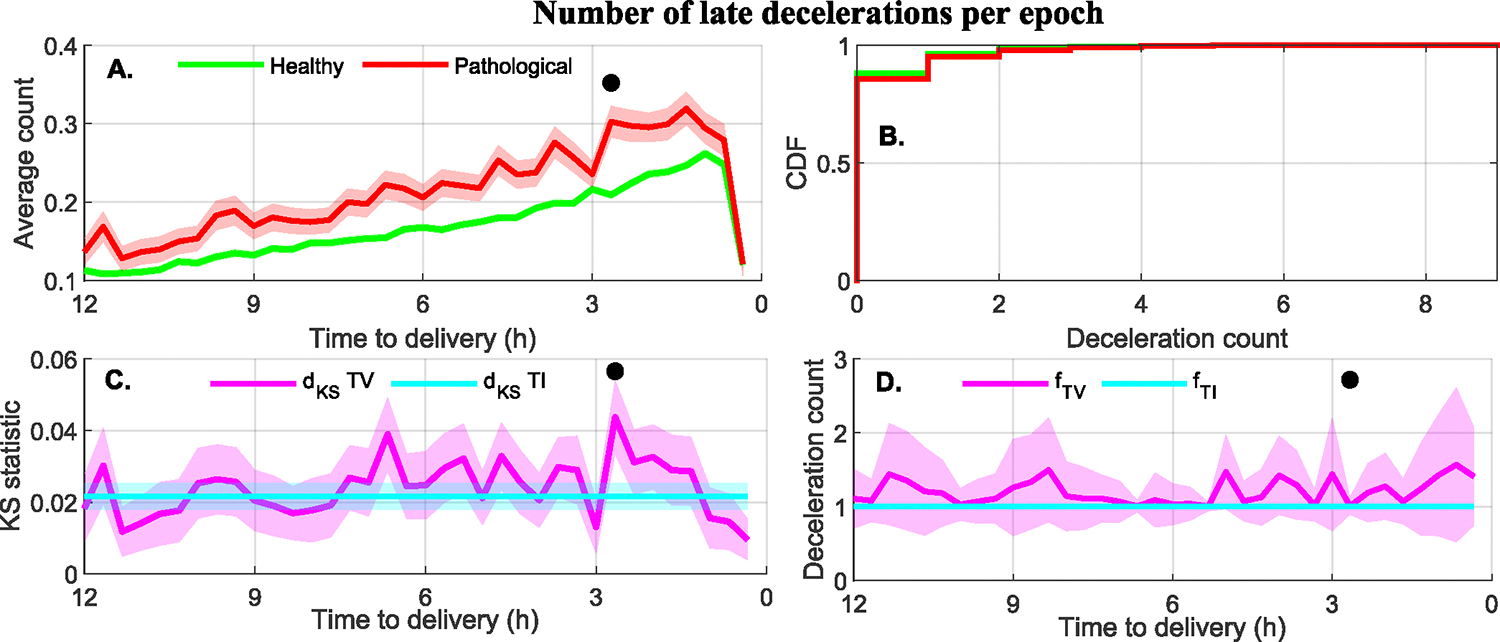
A Type I feature. (A) Average number of late decelerations per epoch for the healthy and pathological (acidosis and HIE) groups (mean ± standard error). (B) The cumulative distribution of the feature during the last 12 hours of labor. (C) Comparison of the TI and TV KS statistic for the binary separability of the healthy and pathological (acidosis or HIE) groups (mean ± standard deviation). (D) Comparison of the feature value for maximum separability using the TI and TV approaches (mean ± standard deviation). All plots showing data as function of the time to delivery also indicate with a black circle those epochs for which the KS test showed statistically significant differences between groups.

Table 4 lists the remaining features categorized according to their association with the outcome:

Type I features: 14 out of 88 features had a TI, but no TV, association with the class. Fig. 7 illustrates a typical Type I feature: the average number of late decelerations per epoch, a discrete variable. Fig. 7A shows the time course of this feature and Fig. 7B is cumulative distribution for each group. Late decelerations were more frequent in the pathological groups than in healthy subjects. Fig. 7C shows that there is little difference between TI and TV d_KS_ values. Finally, Fig. 7D shows that the optimal separation threshold $\overset{ˆ}{f_{TI}}$ for TI is similar to the TV $\overset{ˆ}{f_{TV}}(t)$ across time.

**FIGURE 8.**
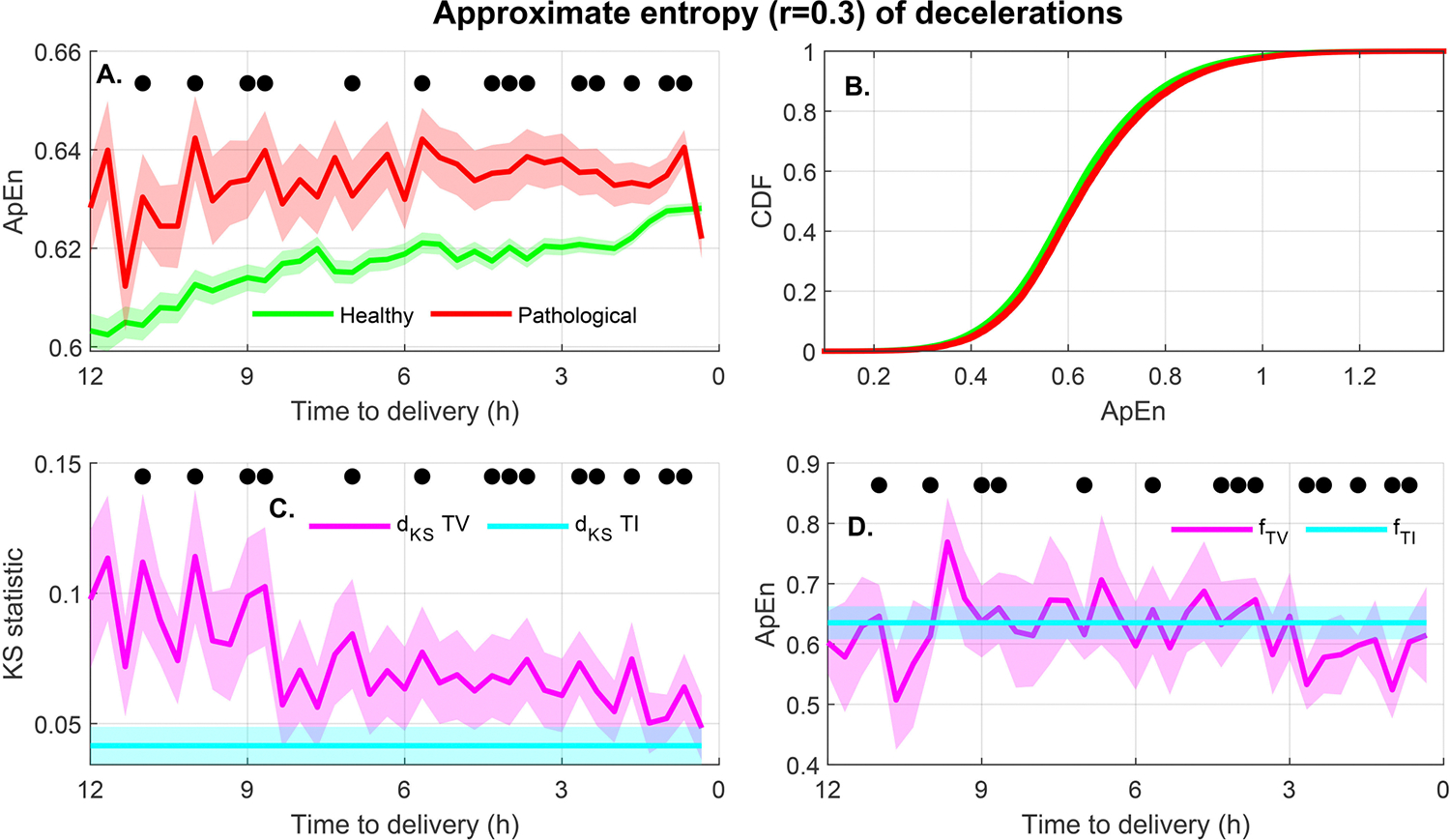
A Type II feature (A) Approximate entropy of the FHR in deceleration events (*r* = 0.3) for the healthy and pathological (acidosis or HIE) groups (mean ± standard error). (B) The cumulative distribution of the feature during the last 12 hours of labor. (C) Comparison of the TI and TV KS statistic for the binary separability of the healthy and pathological groups (mean ± standard deviation). (D) Comparison of the feature value for maximum separability using the TI and TV approaches (mean ± standard deviation). (A-C) Indicate with a black circle those epochs for which the KS test showed statistically significant differences between groups.

Type II features: Nine features had a TV association with the outcome of labor but no TI association. Fig. 8 shows the results for the Type II feature with the highest NMI, the approximate entropy (ApEn) of decelerations. Fig. 8A shows the feature increased with time in the healthy group but changed little in the pathological groups; as a result the trajectories of the healthy and pathological groups converged as labor progressed. There were multiple individual epochs where the KS test detected significant differences. In contrast, the TI cumulative distribution in Fig. 8B showed little difference among groups. Fig. 8C shows that the KS value for the TV features was always higher than the TI. Interestingly, Fig. 8D shows that $\overset{ˆ}{f_{TI}}$ and $\overset{ˆ}{f_{TV}}$ were similar at all times with a value close to 0.63. Thus, as Fig. 8A shows, this threshold would separate the healthy and HIE centers at multiple times. However, due to the convergence of the trajectories, the distance between the centers is much greater for early epochs than for those closer to delivery. Although the optimal threshold does not change with time, its ability to separate the distributions does.

**FIGURE 9.**
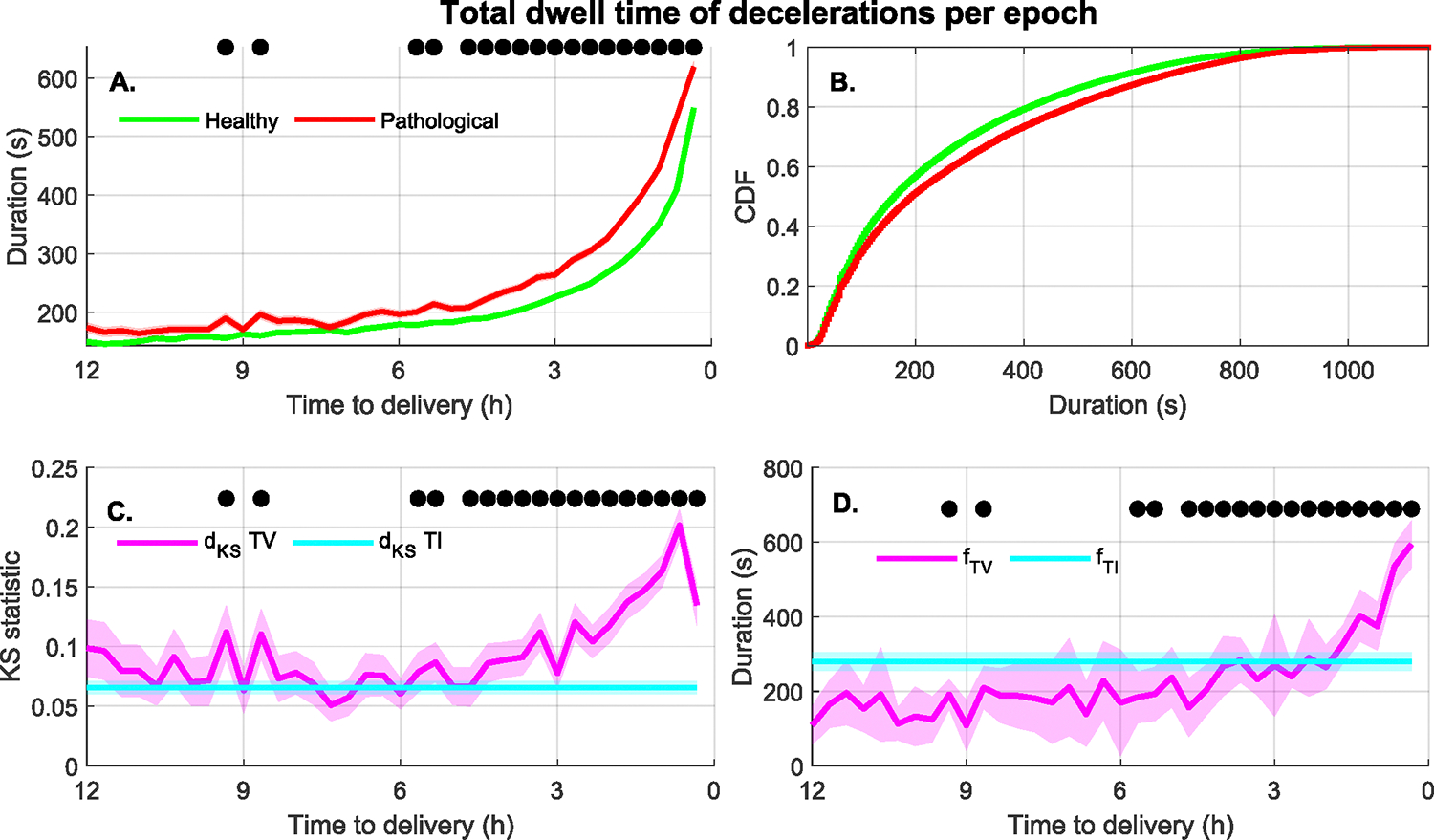
A Type III Feature. (A) Total length of deceleration events per epoch for the healthy and pathological (acidosis or HIE) groups (mean ± standard error). (B) The cumulative distribution of the feature during the last 12 hours of labor. (C) Comparison of the TI and TV KS statistic for the binary separability of the healthy and pathological groups (mean ± standard deviation). (D) Comparison of the feature value for maximum separability using the TI and TV approaches (mean ± standard deviation). All plots showing data as function of the time to delivery also indicate with a black circle those epochs for which the KS test showed statistically significant differences between groups.

Type III features: 17 out of the 88 CTG features were discriminative of the infant groups with a TI approach, and the TV approach further increased their association. Fig. 9A shows the time course of a typical Type III feature, the total duration of deceleration events per epoch. Fig. 9B shows that the TI cumulative distribution differed across groups. Fig. 9A also shows that the distribution of this feature, and the differences between the healthy and pathological groups, varied significantly as labor progressed. Between 12 to 6 hours before delivery, the TI and TV *d*_*KS*_ were similar, as shown by Fig. 9C. However, from 6 hours until delivery, the TV d_KS_ increased. Furthermore, Fig. 9D shows that the optimal separation threshold $\overset{ˆ}{f_{TV}}$ changed with time. Thus, in that example, both the threshold and separability were time varying.

**FIGURE 10.**
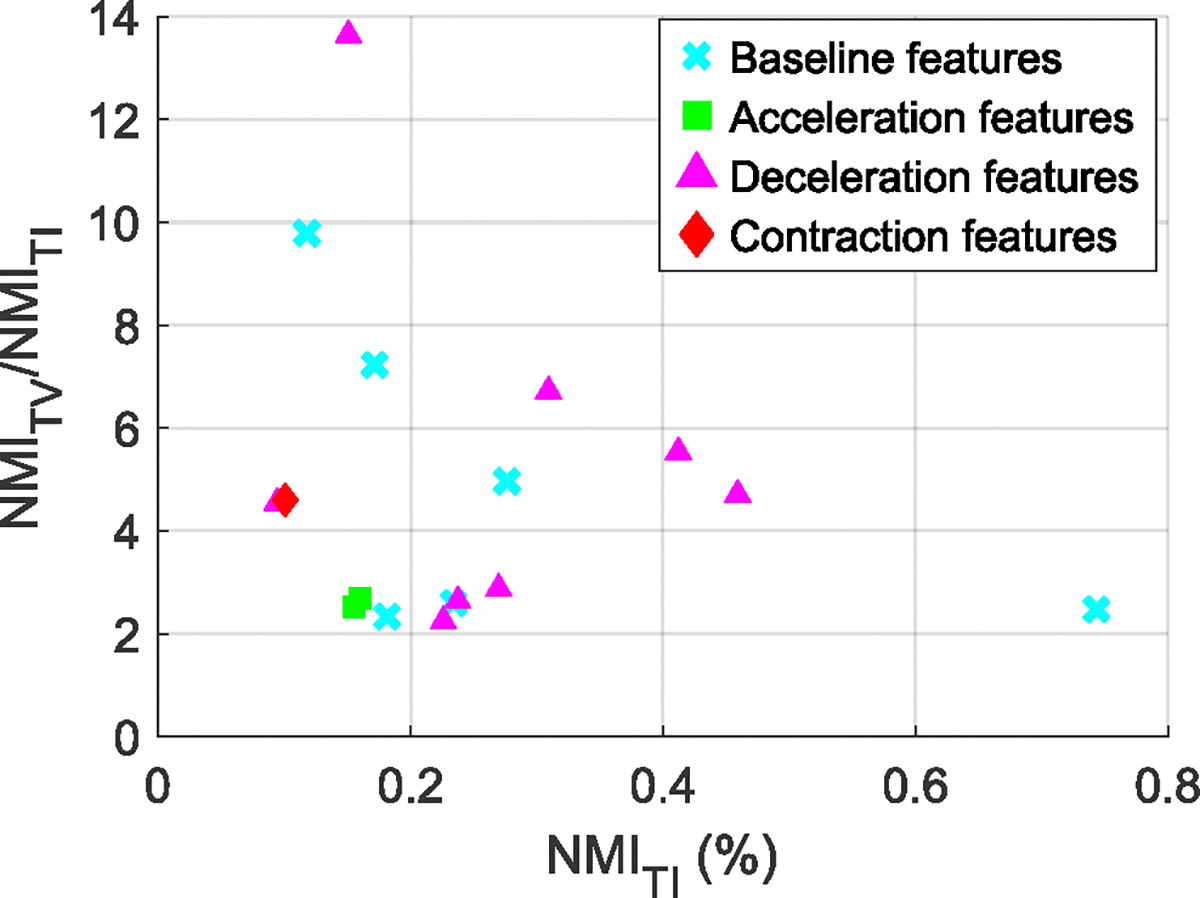
Comparison of the TI NMI with the TV NMI.

Fig. 10 summarizes the ratio between the TV and TI NMI for Type III features. In all cases, the TV ratio was at least twice the TI, demonstrating the significant advantage of the TV approach. Note that Table 6 (Appendix B) reports the NMI values for all significant features.

**FIGURE 11.**
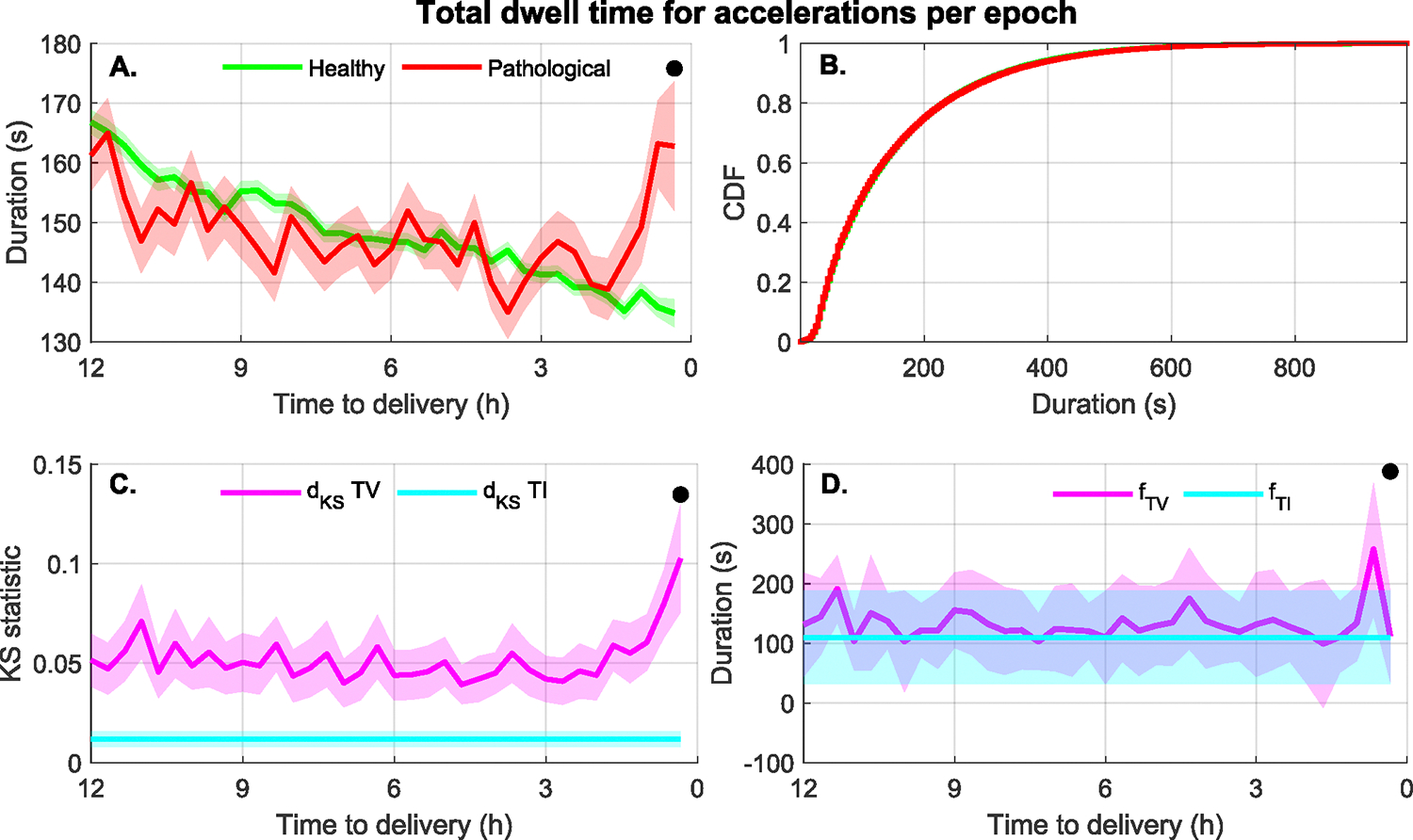
(A) Total length of acceleration events per epoch for each of the healthy and pathological (acidosis and HIE) infant groups (mean ± standard error). (B) The cumulative distribution of the feature during the last 12 hours of labor. The green line showing the cumulative distribution of the feature for infants in the healthy group is hidden by the other curve which overlap with each other. (C) Comparison of the TI and TV KS statistic for the binary separability of the healthy and pathological groups (mean ± standard deviation). (D) Comparison of the feature value for maximum separability using the TI and TV approaches (mean ± standard deviation). All plots showing data as function of the time to delivery also indicate with a black circle those epochs for which the KS test showed statistically significant differences between groups.

Uninformative Features: Table 3 lists the 25 unique FHR feature types that were not informative of the outcome of labor. None of the contraction features were uninformative. From this list, nine features were uninformative when computed exclusively from acceleration events, five features were uninformative when computed from either baseline or acceleration events, four features were uninformative when computed from either acceleration or baseline events, and 7 features were uninformative regardless of the FHR event used for their computation. Thus, a total of 48 feature-event combinations are included in the list of null features. Fig. 11A shows one such feature, the total duration of acceleration events per epoch. It is evident that the values for healthy and pathological groups overlap, and only the last epoch before delivery showed a significant difference. Consequently, the TI cumulative distributions for these groups, shown in Fig. 11B, completely overlap with no differences that could discriminate among groups. In Fig. 11C it appears that the TV values of d_KS_ are larger than the TI values; however, according to the KS test these differences were not large enough to be significant. Finally, Fig. 11D shows that $\overset{ˆ}{f_{TI}}$ and $\overset{ˆ}{f_{TV}}$ were similar, but not useful, since the differences across groups were not significant.

## IV. DISCUSSION

In this study, for a set of 88 intrapartum CTG features proposed in the literature, we analyzed their time dependencies and ability to discriminate the outcome of labor in a large cohort of births. We found that 40 out of 88 features had a significant association with the outcomes. These results, based on the analysis of a large sample of births, support the use of this subset of CTG features as predictors of acidosis and HIE. Importantly, for 26 of these 40 features, their association with outcome increased substantially when accounting for the time to delivery. This strongly indicates that accounting for TTD would improve the performance of classifiers aiming to detect the risk of developing HIE in labor. Finally, we found that 48 out of 88 features were not significantly associated with the outcome of labor. All these features had previously been proposed for use in the detection of fetal hypoxemia. Thus, our results, based on a large sample size, defined features are not likely to be informative. We believe that these findings will help future machine-learning studies to focus on features that are associated with fetal acidosis and an increased risk of HIE.

### A. CHARACTERISTICS OF THE DATASET

In terms of their clinical and demographic characteristics (Table 2) the acidosis and healthy groups showed significant differences in gestational age, maternal age, and maternal weight. However, the effect size of these differences was very small. In contrast, nulliparity was quite different between the groups: 73.05% of HIE resulted from nulliparous pregnancies, while the incidence of nulliparity in the healthy group was 55.76%. Labors in nulliparous pregnancies tend to be longer and more complicated than in subsequent pregnancies. Longer labors carry increased risk of infections and other complications to the mother and fetuses. Thus, it is sensible that nulliparity be more common among cases that developed HIE. Furthermore, the median labor length was almost twice as long in the HIE group than in the healthy group. Labor length was significantly different in the healthy and acidosis groups, and in the healthy and HIE groups.

Signal availability also differed across the three groups. In general, there were a higher proportion of CTG epochs available in the HIE group than in the acidosis and healthy groups. The increased availability of the HIE group could be caused by different factors. For instance, it is possible that towards the end of delivery, clinicians noticed some worrisome patterns in this group and made efforts to improve the monitoring quality leading to more data being available. Whereas for earlier epochs, the increased availability of epochs in the HIE groups may be a consequence of labor length. Another relevant characteristic of the data availability curves is that the amount of data available dropped dramatically during the last hour of labor. This could be explained by several factors. For example, the increased number of contractions plus active pushing might increase signal noise, resulting in an epoch that is rejected for low quality. Another explanation is that there may be increased maternal movement in the last hour of labor, leading to signal disconnections. Despite the differences in signal availability, the number of available healthy and acidosis cases was still large. Thus, we do not consider that a reduction in signal availability in these groups could affect negatively the interpretation or significance of our results.

It is important to note that our study is the first to analyze the associations of CTG features and outcomes of labor on such a large group of HIE cases for as long as the last 12 hours of labor. Furthermore, we were able to differentiate the usability of individual features for each type of FHR event. Previously, this was not feasible for large databases due to the need for manual labelling of such events. It is expected that FHR events contain differentiable information of fetal health during labor. Thus, a segregated analysis of these events could help to better determine the most important properties of these events for fetal assessment.

### B. CLINICAL RELEVANCE OF CTG FEATURES

During labor, uterine contractions are the most common triggers of fetal distress and hypoxemia. Contractions compress the fetal and maternal blood vessels that provide oxygen to the fetus. The fetus responds to the resulting periods of transient hypoxemia with FHR decelerations and peripheral vasoconstriction [35]. These responses are mediated by the sympathetic and parasympathetic branches of the autonomic nervous system [35]. Thus, event-based features such as the number, length, height, and area of the FHR decelerations reflect the FHR fetal response to contractions.

We use other time-domain, frequency-domain, and nonlinear features from adult heart rate variability measures that have been adapted to fetal studies [36]. In adults, these features reflect the sympathetic and parasympathetic control of the heart rate [36]. Thus, Signorini et al. associated time-domain (short-term variability, long-term irregularity, interval index), frequency domain (low-frequency, movement-frequency, and high-frequency power), and nonlinear (approximate entropy) features with autonomic nervous system regulation and fetal distress [37]. Since then, these features have been used in multiple CTG classification studies as predictors of fetal distress in labor [24], [25], [38]. CTG features quantify the activity of the autonomic nervous system and may carry information that is useful to detect fetuses in distress during labor. Thus, to obtain an objective measure of fetal pathology in this study, we tested a comprehensive set of CTG features and determined which were significantly associated with HIE at birth.

### C. TI ASSOCIATION OF CTG FEATURES WITH LABOR OUTCOMES

In this study we used the NMI to quantify associations among variables. Due to its properties, the NMI has been used in classification studies as a model-free method to assess feature importance according to their univariate association with the target variable [19]. Similarly, the conditional mutual information has been used to sequentially select informative and minimally redundant new features for classification purposes [39]. Therefore, increases in the NMI of these features can be understood as an indicator of their potential to enhance the performance of automated CTG classifiers.

Seven of the features we examined were related to the clinical NICHD guidelines in Table 1 and are used clinically to detect infants at risk of fetal compromise during labor [4]. These features were listed in section II–C. We found that six out of these seven features were significantly associated with the infant outcome. The exception was the peak-to-peak FHR variability which showed no significant association with the outcome of labor. The guidelines consider absent peak-to-peak variability as an indicator of increased risk of fetal compromise in labor. However, in our database, only 2.92% of epochs had a peak-to-peak variability below 5 bpm; 0.09% had variability less than 3 bpm. With such low prevalence, it is not surprising that the lower tail of this distribution had insufficient weight to generate a significant NMI.

We reiterate that the wide range of features that have been proposed in other CTG studies [24], [25], [37], have been studied mostly in relation to fetal acidosis rather than to HIE specifically. In our analysis, adding these CTG features to the clinical features led to a total of 88 features. We found that 40 features were significantly associated with an HIE outcome (see Table 4). Most were features of baseline (14) and deceleration (20) events, with only few related to acceleration-based features (3). This demonstrates the importance of decelerations in the identification fetuses at increased risk of developing HIE. Of the group of relevant acceleration-based features, the mean FHR (type I) is a feature influenced by the baseline level. The other relevant features deceleration-to-acceleration transitions (type I); transitions to and from baselines (type III); and acceleration count (type III) all measure the frequency of accelerations per epoch. Thus, our results imply that only the number of accelerations per epoch conveys relevant information about the outcome of labor. This empirical finding agrees with the current clinical assessment of accelerations in the analysis of CTG where only the presence or absence of accelerations is evaluated.

Finally, it is remarkable that all features related to uterine pressure were significantly associated with labor outcomes. These features quantified the frequency of contractions and the recovery time available to the fetus in between contractions. Thus, quantifying contractions and their effect on the FHR may improve our understanding of fetal deterioration towards HIE in labor.

### D. THE IMPORTANCE OF ACCOUNTING FOR TIME

During labor, the risk of hypoxia increases progressively up to the time of delivery [40]. Studies in animal models have demonstrated the evolution of FHR patterns with labor-like umbilical cord occlusions [41]. However, few studies have examined the temporal evolution of FHR features in human cases of HIE [4]. We found that all 88 features we examined were significantly associated with TTD and so were time varying.

To evaluate the role of the TV behavior in the discrimination across groups, we examined the conditional NMI between features and infant groups with respect to the TTD. Twenty-six of the 40 features associated with outcome had significant conditional associations. This indicates that a classifier that uses TV CTG features would perform better than one using TI features.

For example, as Fig. 9 shows, a threshold of 250 seconds for the deceleration dwell time would separate the healthy and HIE groups at three hours before delivery. However, this threshold would not be useful one hour before delivery. In contrast, approximate entropy of the deceleration segments, shown in Fig. 8A, behaved differently. In this case, the center of the distribution of the pathological group did not vary much during the last 12 hours of labor, whereas the distribution of the healthy group evolved with time and converged towards the pathological distribution as delivery approached. As a result, the best threshold to separate the distributions did not change with time as shown in Fig. 8D. However, the separability associated with this threshold was greater at earlier times than closer to delivery (Fig. 8C). These two examples illustrate the advantages of time-varying classification models. Thus, in some cases the decision thresholds may continuously evolve with time as in Fig. 9D. In other cases, the threshold may not change but its usefulness does. Thus, time-varying classification models should learn how the distributions of features in labor evolve but also when these diverge the most and are most useful for classification.

Our results suggest that features of Types II and III would be most useful for the detection of HIE if the decision rules were TV. Previous studies have evaluated the varying incidence of certain abnormal FHR patterns during labor [42], [43]. However, our study is the first to quantify the evolution of these features with time. We believe that the definition of an ‘abnormal’ pattern requires understanding its evolution; as described above, what is abnormal three hours before delivery might not be abnormal one hour before delivery.

The benefit of a time-varying analysis of FHR is substantial. Fig. 9C shows, for deceleration dwell time, that the TV KS statistic doubles during the last two hours of labor. Also, Fig. 10 shows that conditional NMI is about four times larger than the TI NMI. In consequence, accounting for time could result in better classification performances to detect fetuses at increased risk of HIE in labor.

### E. LIMITATIONS AND FUTURE WORK

We have shown that all features varied with the TTD. However, it is not possible to apply this insight prospectively since TTD is only available after delivery. Thus, future studies should explore other variables to define the temporal evolution of intrapartum FHR that are available during labor. Some alternative variables to explain FHR evolution could be the time from labor onset, or the cumulative number and length of contractions or decelerations. The time from labor onset quantifies how long the fetus has endured labor, while the cumulative length of contractions and decelerations quantify the total length of hypoxemic events that the fetus has endured. Future studies could quantify the association between CTG features and the outcome of labor in the context of these new variables and compare them to the results presented here. A surrogate time variable that outperforms or matches the NMI obtained with TTD would greatly improve clinical utility.

The purpose of aligning the FHR with respect to the time of delivery was to standardize its evolution in the last hours of labor. This alignment cannot be applied to Caesarean deliveries, where the time of delivery does not represent the natural end of labor. We addressed this limitation by selecting only vaginal deliveries in our study. Although this restrictive selection halved the number of available HIE cases, we still had enough data to reject most null hypotheses in our study. It is important to note that this is a limitation that would be removed by using an alternative time variable for prospective use.

Our analysis showed which features would be relevant in univariate classifiers, yet machine-learning models rarely consider single features in isolation. Instead, they combine multiple features to map associations that might be difficult to infer from univariate assessments. We would expect that such interactions among features will translate to greater improvements in the prediction of the risk of developing HIE than those seen solely from univariate analysis. To better utilize these features, future machine-learning classifiers should first perform feature importance studies where they also explore inter-feature dependencies to reduce model redundancy. To date, studies based on stationary rules for the detection of severe fetal complications, including HIE, have shown promising detection performance [12], [13], [24]. We believe that this initial performance can be further improved by accounting for the temporal evolution of the CTG features as we recently showed in preliminary studies [44], [45]. We also believe that understanding the trajectory of the clinically relevant features with TV distributions could be fundamental to updating and improving current clinical guidelines.

Finally, it is also important to note that our study focused only on the evolution of CTG features over long periods of time. The choice of epoch length, 20 minutes in this study, affects the estimation of the CTG features. Using shorter epochs would allow us to be more sensitive to local changes but would also increase the variability of the estimate. In contrast, longer epochs would yield more robust estimates of the features but the differences across classes may be less obvious. As we can see from our results, 20-minute epochs were short enough to show the differences across classes and time-dependency without large variability. Future studies could evaluate the discriminability of CTG features as a function of the epoch length. Furthermore, our study made no attempts at quantifying the local dynamic FHR response to uterine contractions. In a previous study, we showed that the FHR signal responds to changes in uterine contraction following a nonlinear dynamic system [46]. These causal nonlinear dynamics can also be interpreted as local changes in the stationarity of the signal. Quantifying and evaluating these local changes can provide important information about the immediate fetal response to stressful stimuli. Such response reflects the health of the fetal autonomic nervous system and peripheral chemoreflex [35]. Future studies could use features that reflect local changes, such as spectrograms, or quantify the evolution of the parameters of the system that modulates FHR in response to uterine contractions.

### F. IMPLICATIONS FOR CLASSIFICATION STUDIES

The main objective of machine- and deep-learning CTG classifiers is to detect fetuses that are progressing towards acidosis and HIE during labor. Such predictions could lead to timely clinical interventions, i.e., emergency Caesarean deliveries, which could mitigate or prevent the serious consequences of acidosis. Despite recent advances in the field, automated CTG studies have not yet achieved sufficient performance to be used clinically [47]. This is partly due to concerns about increasing the number of unnecessary Caesarean deliveries [48]. Another reason could be that most of these classifiers used signals recorded close to delivery when clinical interventions are not likely to be useful.

Thus, one machine-learning study achieved over 80% sensitivity in the detection of cases of fetal acidosis (pH ≤ 7.05) with a specificity above 85% but used CTG features extracted during the last 30 minutes before delivery [49]. Similarly, another study that used convolutional neural networks to detect fetal acidosis (pH ≤ 7.05) obtained a sensitivity of 56.15% for a specificity of 96.51% but used only the last 15 minutes of labor [50]. Thus, their ability to detect fetuses at risk earlier in labor, when interventions are possible, remains questionable [51].

The present study builds on some preliminary results which showed that the properties of the CTG signals are time-varying [52], [53]. Here, we used empirical methods to show that all features examined were time-varying. Moreover, for many, accounting for the time-variation improved their discriminability. Some deep-learning studies have used similar features to those tested in this study [54]. Other studies used end-to-end approaches, where the network was expected to learn the features of the signal for classification [13]. End-to-end approaches do not use the CTG features we examined explicitly. However, we found that all the time-domain, frequency-domain, and nonlinear features of CTG signals are time-varying. Thus, it is reasonable to hypothesize that any useful deep-learning classifier, including one that is end-to-end, must account for time variability.

We note that some classification studies have examined CTG features at different labor stages. Thus, one study focused only on the first minutes of labor [14], a second study trained its classifier using data from both the end of the 1st stage and the end of the 2nd stage of labor [13], and a third study used only data from the end of 1st stage of labor [55]. Although useful, the resulting models can only be valid for short periods of labor. We believe that future classification studies should deal with the continuum of intrapartum CTG evolution. Thus, future classifiers could include time variables, such as time since labor onset, as additional classification features. In this approach, the classifiers could learn the joint distribution of CTG features and time, which would allow them to account for the evolution of the features. Another approach would be to make the classifier parameters time-varying. Thus, the classification model would use time-varying decision rules. A third approach would be to develop multiple classifiers for different times throughout labor.

## V. CONCLUSION

Our results showed that all 88 CTG features have TV properties. We also showed that 48 features had no association with the outcome of labor and so are unlikely to be useful for classification. Of the 40 features that had significant associations with the outcome of labor, 26 of these had larger TV associations that were also significant. We believe that these results should provide guidance to future classification studies by focusing on those features that were significantly associated with labor outcomes. In addition, our results indicate that accounting for the temporal evolution of FHR should improve the detection of fetuses at increased risk of intrapartum HIE.

## Figures and Tables

**TABLE 1.** Clinical classification of FHR patterns.

Category	Definition	Interpretation
Category I: Normal	FHR tracings must have all the following: • Baseline level in the 110 – 160 beats-per-minute (bpm) range. • Baseline peak-to-peak variability in the 6 – 25 bpm range. • Absence of late or variable decelerations. • Present or absent early decelerations. • Present or absent accelerations.	When observed, these tracings are predictive of normal acid-base levels. No special care is required.
Category II: Indeterminate	All FHR tracings that do not fall in Category I or Category III.	These patterns are not predictive of good or bad outcomes. They required reevaluation and considering other clinical factors that might be relevant to the fetus.
Category III: Abnormal	FHR has absent peak-to-peak variability in the baseline and at least one of the following: • Recurrent late decelerations. • Recurrent variable decelerations. • Baseline level below 110 bpm. Alternatively, FHR that shows a sinusoidal pattern with no other type of visual variability.	These patterns require immediate action. Suggested approaches include supplying oxygen to the mother, changing maternal position, removing labor stimulation, and treating maternal hypotension.

Definition and interpretation of the categories proposed by the NICHD to classify FHR patterns [4].

**TABLE 2.** Demographic and clinical characteristics of participants.

	Healthy *n* = 22,903	Acidosis *n* = 1,912	HIE *n* = 167	*p*1	*p*2
**Birth weight (kg)**	3.39 (3.07 – 3.72)	3.36 (3.07 – 3.68)	3.41 (3.01 – 3.79)	0.0907 (−0.047)	0.9197 (0.002)
**Gestational age (weeks)**	39.71 (38.86 – 40.57)	40.0 (39.0 – 40.71)	40.0 (39.0 – 40.57)	**<0.0001 (0.124)**	0.3869 (0.040)
**Maternal age (years)**	31 (27 – 35)	30 (26 – 34)	30 (27 – 34.75)	**<0.0001 (−0.171)**	0.2619 (−0.067)
**Maternal weight (kg)**	78.9 (70.3 – 90.3)	80.6 (71.7 – 92.1)	81.6 (72.7 – 96.45)	**0.0001 (0.083)**	**0.0054 (0.244)**
**Nulliparity**	12,771 (55.76%)	1,305 (68.25%)	122 (73.05%)	**<0.0001**	**<0.0001**
**Length of labor (h)**	11.25 (6.15 – 18.02)	13.33 (7.87 – 21.0)	19.2 (11.0 – 27.52)	**<0.0001 (0.211)**	**<0.0001 (0.782)**

Median (interquartile range) or subject count (percentage) of key characteristics for the three groups. The Mann-Whitney U test was used on continuous variables and the *X*^2^ test on categorical data. The column ***p*1** reports the *p*-values of comparing the acidosis group to the healthy group, and ***p*2** reports the comparison of the HIE group to the healthy group. All *p*-values were corrected using the BH method. The numbers in brackets in the ***p*1** and ***p*2** columns are Cohen’s *d* effect size.

**TABLE 3.** List of CTG features not associated with the infant groups.

Feature name	B	A	D
Delta	X	X	X
Low frequency (LF) power		X	X
Movement frequency (MF) power		X	X
High frequency (HF) power	X	X	X
LF/(MF+HF) ratio		X	X
Acceleration capacity	X	X	X
Approximate entropy (r=0.1)	X	X	
Approximate entropy (r=0.2)		X	
Approximate entropy (r=0.3)	X	X	
Correlation dimension	X	X	
Deceleration capacity	X	X	X
Deceleration reserve		X	
Hurst exponent		X	
Interval index		X	
Long term irregularity	X	X	X
Lyapunov exponent		X	
Sample entropy (r=0.1)		X	X
Sample entropy (r=0.2)	X	X	
Sample entropy (r=0.3)	X	X	
Short term variability	X	X	X
Standard deviation (peak-to-peak variability)	X	X	X
Dwell time (total length in one epoch)		X	
Number of transitions to decelerations		X	
Area		X	
Height		X	

FHR features extracted from the baseline (B), acceleration (A), and deceleration (D) events. The X marks indicate which features were not useful from each event type.

**TABLE 4.** List of CTG features not associated with the infant groups.

Feature name	B	A	D	C
Low frequency (LF) power	III			
Movement frequency (MF) power	III			
LF/(MF+HF) ratio	II			
Approximate entropy (r=0.1)			III	
Approximate entropy (r=0.2)	II		I	
Approximate entropy (r=0.3)			II	
Correlation dimension			III	
Deceleration reserve	I		I	
Hurst exponent	I		III	
Interval index	I		II	
Lyapunov exponent	II		II	
Mean FHR (level)	III	I	I	
Sample entropy (r=0.1)	II			
Sample entropy (r=0.2)			II	
Sample entropy (r=0.3)			II	
Slope	I			
Dwell time (total length in one epoch)	III		III	I
Resting time (total length in one epoch)				I
Number of transitions to accelerations	III		I	
Number of transitions to baselines		III	III	
Number of transitions to decelerations	III			
Area			I	
Height			I	
Number of variable decelerations			III	
Number of late decelerations			I	
Number of decelerations that are neither variable nor late			III	
Total event count per epoch		III	III	III

FHR features extracted from the baseline (B), acceleration (A), deceleration (D) and contraction (C) events. Type I features had only a time invariant association; Type II had only a time-varying association, Type III had both time invariant and time-varying associations.

## Data Availability

The datasets generated and analyzed during the current study are not publicly available due to patient privacy policies. Unidentified cardiotocography (CTG) records may be available for research but require submitting a study proposal. The CTG data cannot leave KPNC firewalls under any circumstances. Interested parties might follow these steps: (1) submit a proposal to KPNC Division of Research that includes the author MK (or his designate) as a co-investigator, (2) the proposal will be reviewed and approved by the KPNC Institutional Review Board for the Protection of Human Subjects, and (3) a Data Use Agreement between the interested parties and the Division of Research will be executed. The interested parties must commit to research use only, with no commercial use of the datasets.

## APPENDIX A

### LIST OF CTG FEATURES

In this study we included a comprehensive set of time-domain, frequency-domain, and nonlinear CTG features previously proposed in the literature. We also included features that quantified the sequential interaction between different types of CTG patterns within each epoch. These features are described in the next subsections. The source code used to extract all these features was described in more detail in our methods paper [20]. A complete list of all features extracted from each CTG event is provided in Table 5.

#### A. EVENT DESCRIPTIVE FEATURES

These features quantified properties specific to the CTG events and their sequential interactions. These features were the following:

- The event dwell time described the total duration of each event within each epoch. We computed the dwell time for baseline, acceleration, deceleration, and contraction events.
- Resting time was specific to the UP signal. This feature described the total duration of the epoch where no contractions were present.
- Number of transitions between FHR events counted the times that baseline, acceleration, and deceleration events transitioned into a different event within each epoch.
- Event count per epoch counted the total number of times that acceleration, deceleration, and contraction events were present in one epoch without considering from which event they transitioned.
- Deceleration type count grouped the deceleration count by type. We counted how many decelerations in one epoch were late decelerations, or variable decelerations; we also counted how many decelerations were neither late nor variable.
- Area of the acceleration and deceleration summed the total area of these events within each epoch.
- Maximum height gave an upper estimate of the deviation of acceleration and deceleration events from baseline in one epoch.
- Average slope was measured from a linear fit of baseline events.

In total, 22 event descriptive features were extracted from the CTG signals.

#### B. TIME-DOMAIN FEATURES

Other time-domain features evaluated were:

**TABLE 5.** List of all extracted CTG features.

N	Feature name	B	A	D	C
**1**	Dwell time (total length in one epoch)	✓	✓	✓	✓
**2**	Resting time (total length in one epoch)				✓
**3**	Number of transitions to baselines		✓	✓	
**4**	Number of transitions to decelerations	✓	✓		
**5**	Number of transitions to accelerations	✓		✓	
**6**	Total event count per epoch		✓	✓	✓
**7**	Number of late decelerations			✓	
**8**	Number of variable decelerations			✓	
**9**	Number of decelerations that are neither variable nor late			✓	
**10**	Area		✓	✓	
**11**	Height		✓	✓	
**12**	Slope	✓			
**13**	Mean FHR (level)	✓	✓	✓	
**14**	Peak-to-peak variability (standard deviation)	✓	✓	✓	
**15**	Short-term variability	✓	✓	✓	
**16**	Long-term irregularity	✓	✓	✓	
**17**	Delta	✓	✓	✓	
**18**	Interval index	✓	✓	✓	
**19**	Low frequency (LF) power	✓	✓	✓	
**20**	Movement frequency (MF) power	✓	✓	✓	
**21**	High frequency (HF) power	✓	✓	✓	
**22**	LF/(MF + HF) ratio	✓	✓	✓	
**23**	Approximate entropy (r=0.1)	✓	✓	✓	
**24**	Approximate entropy (r=0.2)	✓	✓	✓	
**25**	Approximate entropy (r=0.3)	✓	✓	✓	
**26**	Sample entropy (r=0.1)	✓	✓	✓	
**27**	Sample entropy (r=0.2)	✓	✓	✓	
**28**	Sample entropy (r=0.3)	✓	✓	✓	
**29**	Correlation dimension	✓	✓	✓	
**30**	Lyapunov exponent	✓	✓	✓	
**31**	Hurst exponent	✓	✓	✓	
**32**	Acceleration capacity (AC)	✓	✓	✓	
**33**	Deceleration capacity (DC)	✓	✓	✓	
**34**	Deceleration reserve	✓	✓	✓	

Complete list of CTG features extracted from the baseline (B), acceleration (A), deceleration (D) and contraction (C) events. The checkmarks indicate which features were computed from each FHR and UP event.

- Mean value of the FHR within baseline, acceleration, and deceleration events, was measured after being filtered by a low-pass filter with cut-off frequency at 0.03 Hz. The filtering removed the effect of low-frequency trends such as the baseline slope. In the case of the baseline mean FHR, this feature is also known as the FHR baseline level.
- Standard deviation of the baseline, acceleration, and deceleration events was measured after being filtered by a high-pass filter with cut-off frequency at 0.03 Hz. If we assume a normal distribution for the FHR, the baseline peak-to-peak variability could be approximated as the 2.5th - 97.5th percentile range of the FHR distribution, which is directly proportional to its standard deviation.
- Short-term variability quantifies the variation of the interval between consecutive beats.
- Long-term irregularity quantifies slow variations in the FHR.
- Delta is a feature that quantifies the maximum range of the FHR in one-minute windows.
- Interval index compares the short-term variability to the standard deviation of the FHR.

A total of six time-domain features were extracted from each FHR event type, resulting in a total of 18 features.

#### C. FREQUENCY-DOMAIN FEATURES

These features quantified the power that the signal contained in previously defined frequency bands:

- Low-frequency (LF) power is the total power of the signal in the 30 – 150 mHz band.
- Movement frequency (MF) power is the total power of the signal in the 0.15 – 0.5 Hz band.
- High frequency (HF) power is the total power that the signal contains in the 0.5 – 1 Hz band.
- The LF/(MF + HF) ratio quantifies the interaction between these frequency bands.

A total of four frequency-domain features were extracted from each FHR event type, resulting in a total of 12 features.

#### D. NONLINEAR FEATURES

Other nonlinear features that were previously proposed in the literature were extracted from our CTG database:

- The approximate entropy is a measure of signal regularity. It compares segments of the signal with itself to quantify the randomness of the signal. We computed the approximate entropy when the *r* parameter was set to 0.1, 0.2, and 0.3.
- The sample entropy is similar to the approximate entropy. However, its calculation is independent of signal length which makes it useful to compare across signals with different number of samples. We computed the sample entropy when the *r* parameter was set to 0.1, 0.2, and 0.3.
- The correlation dimension is another measure of signal randomness, but this quantifies the fractality of a signal.
  **TABLE 6.** NMI values for all significant CTG features.
  N	Event	Feature name	*NMI_F^i^_*(*F^i^*; *TTD*)	*NMI_C_*(*C*; *F^i^*)	*NMI_C_*(*C*; *F^i^*|*TTD*)
  **23**	D	Approximate entropy (r=0.1)	1.52	0.41	2.28
  **1**	D	Dwell time (total length in one epoch)	2.99	0.46	2.16
  **31**	D	Hurst exponent	1.64	0.31	2.08
  **29**	D	Correlation dimension	0.85	0.15	2.06
  **25**	D	Approximate entropy (r=0.3)	0.32	–	2.04
  **27**	D	Sample entropy (r=0.2)	0.52	–	2.00
  **28**	D	Sample entropy (r=0.3)	0.64	–	1.96
  **18**	D	Interval index	0.75	–	1.89
  **13**	B	Mean FHR (level)	0.68	0.74	1.84
  **30**	D	Lyapunov exponent	0.78	–	1.83
  **30**	B	Lyapunov exponent	0.79	–	1.65
  **1**	B	Dwell time (total length in one epoch)	2.66	0.28	1.37
  **26**	B	Sample entropy (r=0.1)	0.22	–	1.28
  **24**	B	Approximate entropy (r=0.2)	0.45	–	1.25
  **19**	B	Low frequency (LF) power	0.79	0.17	1.24
  **22**	B	LF/(MF + HF) ratio	0.18	–	1.23
  **20**	B	Movement frequency (MF) power	0.09	0.12	1.15
  **6**	D	Total event count per epoch	5.81	0.27	0.78
  **3**	D	Number of transitions to baselines	5.25	0.24	0.63
  **4**	B	Number of transitions to decelerations	5.15	0.23	0.61
  **13**	D	Mean FHR (level)	0.52	0.60	–
  **9**	D	Number of decelerations that are neither variable nor late	4.54	0.23	0.51
  **6**	C	Total event count per epoch	0.84	0.10	0.46
  **6**	A	Total event count per epoch	0.57	0.16	0.43
  **8**	D	Number of variable decelerations	6.38	0.09	0.43
  **5**	B	Number of transitions to accelerations	0.83	0.18	0.42
  **3**	A	Number of transitions to baselines	1.10	0.16	0.39
  **13**	A	Mean FHR (level)	0.52	0.38	–
  **10**	D	Area	3.85	0.34	–
  **24**	D	Approximate entropy (r=0.2)	0.54	0.29	–
  **34**	D	Deceleration reserve	0.43	0.19	–
  **11**	D	Height	2.11	0.17	–
  **12**	B	Slope	0.79	0.17	–
  **34**	B	Deceleration reserve	0.52	0.14	–
  **7**	D	Number of late decelerations	0.76	0.10	–
  **31**	B	Hurst exponent	1.10	0.10	–
  **1**	C	Dwell time (total length in one epoch)	0.58	0.09	–
  **2**	C	Resting time (total length in one epoch)	0.63	0.08	–
  **18**	B	Interval index	0.46	0.08	–
  **5**	D	Number of transitions to accelerations	2.01	0.03	–
  NMI estimates for all significant CTG features. The first column indicates the index of the feature, which matches those in Table 5. The second column indicates the event that was the source for the feature: baseline (B), acceleration (A), deceleration (D), and contraction (C). The third column indicates the name of the feature. The fourth column shows the NMI between the features and the time to delivery. The fifth column shows the time-invariant NMI between the features and the outcome of labor. Lastly, the sixth column shows the NMI between features and the outcome of labor, conditioned to the time to delivery. Whenever an NMI estimate was not found to be significant, it was indicated with a blank space (-). The features in this table were sorted in descending order of the maximum information that they provide on the outcome of labor (the maximum between the two last columns).
- The Lyapunov exponent assesses the dependence of the trajectories within a signal on the initial conditions; it is another measure of regularity.
- The Hurst exponent is another measure of signal fractality. Unlike the correlation dimension that focuses on the distribution of a signal, or the Lyapunov exponent that focuses on short-term trajectories, the Hurst exponent looks at the long-term behavior of the signal.
- The phase rectified signal averaging (PRSA) analysis consists of aligning small windows of a signal that have an increasing or decreasing tendency. The PRSA results from the averaging of these aligned windows. The acceleration capacity (AC) is obtained from the assessment of the PRSA when the windows have an increasing tendency. In contrast, the deceleration capacity (DC) is obtained from the assessment of the PRSA when the windows have a decreasing tendency. Finally, the deceleration reserve *DR* = |*DC*| − *AC* can be obtained to compare the dominant tendency of the signal.

A total of 12 time-domain features were extracted from each FHR event type, resulting in a total of 36 features.

## APPENDIX B

### LIST OF SIGNIFICANT FEATURES

Table 6 lists all significant features and the estimated NMI for each of them. First, the table reports ${NMI}_{F^{i}}\left(F^{i};TTD\right)$, the amount of information that the TTD provides on each feature. Table 6 also shows ${NMI}_{C}\left(C;F^{i}\right)$ and ${NMI}_{C}\left(C;F^{i}|TTD\right)$, the NMI between the features and the outcomes of labor independent and dependent on time respectively. These last two columns show blank spaces in those cases where the NMI were not significant. The table was sorted in descending order of the maximum NMI that each feature yields with the outcome of labor (the last two columns).
